# Post-translational modifications of CDK5 and their biological roles in cancer

**DOI:** 10.1186/s43556-021-00029-0

**Published:** 2021-07-20

**Authors:** Gui-Bin Gao, Yue Sun, Run-Dong Fang, Ying Wang, Yang Wang, Qing-Yu He

**Affiliations:** 1 MOE Key Laboratory of Tumor Molecular Biology and Key Laboratory of Functional Protein Research of Guangdong Higher Education Institutes, Institute of Life and Health Engineering, College of Life Science and Technology, Jinan University, Guangzhou, 510632 China; 2 Institute of Chinese Medical Sciences and State Key Laboratory of Quality Research in Chinese Medicine, University of Macau, Avenida da Universidade, Taipa, Macao SAR China

**Keywords:** CDK5, Posttranslational modifications, Cancer

## Abstract

Post-translational modifications (PTMs) of Cyclin-dependent kinase 5 (CDK5) have emerged as important regulatory mechanisms that modulate cancer development in patients. Though CDK5 is an atypical member of the cyclin-dependent kinase family, its aberrant expression links to cell proliferation, DNA damage response, apoptosis, migration and angiogenesis in cancer. Current studies suggested that, new PTMs on CDK5, including S-nitrosylation, sumoylation, and acetylation, serve as molecular switches to control the kinase activity of CDK5 in the cell. However, a majority of these modifications and their biological significance in cancer remain uncharacterized. In this review, we discussed the role of PTMs on CDK5-mediated signaling cascade, and their possible mechanisms of action in malignant tumors, as well as the challenges and future perspectives in this field. On the basis of the newly identified regulatory signaling pathways of CDK5 related to PTMs, researchers have investigated the cancer therapeutic potential of chemical compounds, small-molecule inhibitors, and competitive peptides by targeting CDK5 and its PTMs. Results of these preclinical studies demonstrated that targeting PTMs of CDK5 yields promising antitumor effects and that clinical translation of these therapeutic strategies is warranted.

## Introduction

CDK5 is an atypical member of cyclin-dependent kinases (CDKs) located in chromosome 7q36, which shares large proportion of amino acid sequence identity with other CDK members [1, 2]. Its activity is required for cell cycle, transcriptional initiation and metabolic cascades [3, 4]. However, unlike other CDKs, the activation of CDK5 requires specific activators including p35 (Cyclin-Dependent Kinase 5 regulatory subunit 1, CDK5R1) and p39 (Cyclin Dependent Kinase 5 regulatory subunit 2, CDK5R2) that are structurally different from canonical cyclins [5]. CDK5 was firstly discovered in the bovine brain [6] for its function in neuronal development and differentiation [7]. In past decades, the role of CDK5 in cancer development was emerging, which involves in cancer metastasis, proliferation, angiogenesis and chemoresistance. In this review, we introduced the functions of CDK5 in multiple cancer malignant transformation, focusing on various post-translation modifications (PTMs) on CDK5-mediated biological progresses. The advancements of recent investigations on small molecules and competitive inhibitors that specifically target CDK5 or CDK5/p35 complex were also discussed.

## The structure and basic function of CDK5

**Fig. 1:**
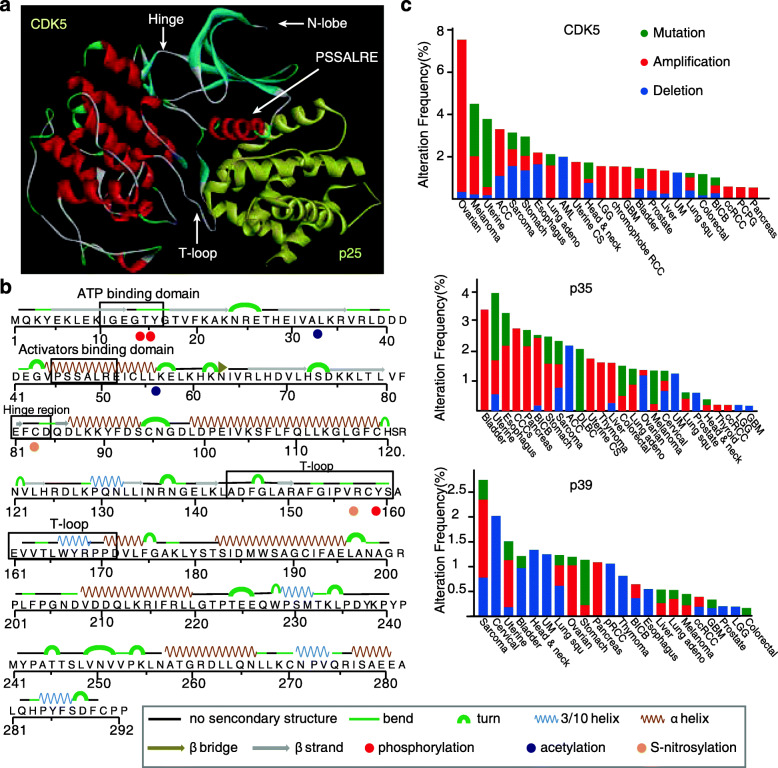
The structure and amino acid sequence of CDK5 and its expression in cancers. **a** The interaction between CDK5 and p25 (yellow). CDK5 structurally contains an ATP binding domain, activators binding domain, hinge region, PSSALRE helix and T-loop. The N lobe mainly contains 5 β-sheets and C lobe includes 4 α-helices. **b** CDK5 amino acid sequence contains β-bridge, bend, turn, β-strand and a-helix sequence. The PTM sites are labelled with colors: phosphorylation, red; acetylation, blue; S-nitrosylation, yellow. **c** The genomic alterations including mutation, amplification and deletion of CDK5 as well as p35 and p39 were analyzed in clinical samples from cBioPortal (www.cbioportal.org)

Similar to other CDK members, CDK5 structurally contains N-lobe, C-lobe, ATP binding domain, activators binding domain, hinge region, PSSALRE helix and T-loop **(**Fig. 1a**)**. The N-lobe mainly contains 5 β-sheets, while C lobe includes 4 α-helices. The PSSALRE helix and the DGF motif in T-loop forming a stereo-structure in the surface of CDK5 are critical for activator binding, such as p35 or p25 [5]. The binding of p25 on CDK5 can effectively tether the conformation and alter the activation loop to an active state **(**Fig. 1b**)**. In addition, the ATP-binding domain located on the surface of CDK5 can receive ATP for activation by regulating the crossing of DFG motif to PPD motif; during this progress, hinge region can form hydrogen bonds with the ATP cleft**.** The functions of these domains can be controlled by various PTMs. For example, in ATP-binding domain, phosphorylation of Thr14 and Tyr15 controlled by the dual specificity kinase Wee1 and Myt1 can influence CDK5 activity [8], whereas phosphorylation of Ser159 in the T-loop of CDK5 contributes to its specific binding to p35 for activation [9]. S-nitrosylation of Cys83, a critical amino acid within the ATP-binding pocket, plays an essential role in regulating the kinase of CDK5 **(**Fig. 1b**)**. It is clear that PTMs occurred on the different domains play important roles in switching the molecular function of CDK5.

## The activators of CDK5

**Fig. 2:**
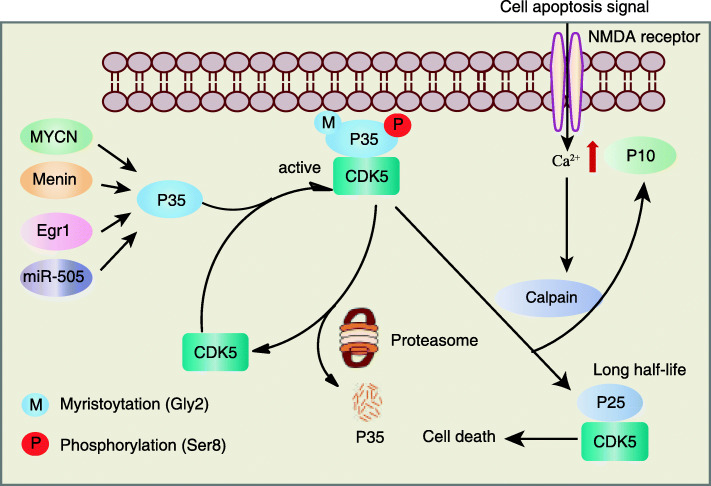
The mechanism of CDK5 activation. The expression of p35 can be transcriptionally regulated by upstream regulators N-Myc, Menin, EGR1 and miR-505. Myristoylation on Gly2 of p35 determines its affinity to cell membrane, which signals for proteasomal degradation. Activated CDK5 phosphorylates p35 on Ser8 to sustain its cytoplasmic localization. When the cell faces with the death signal, the N-methyl-D-aspartate (NMDA) receptor on the cell membrane can be activated, enhancing the uptake of calcium for calpain activation. Calpain has proteolytic activity to cleave p35 into p25 fragment, a more stable protein than p35. This cleavage leads to the translocation of CDK5/p25 complex into the nucleus and prolongs the activation time of CDK5, which induces the pathological signal pathway of cell death

CDK5 was recognized to be activated by non-cyclin proteins p35 and p39. p35 is the first regulatory subunit found to bind with CDK5 and control its activity. It is a short half-life protein that can be rapidly degraded through proteasomes [10–13]. When suffered from harmful stimulation, the calpain protease can induce the cleavage of p35 to produce p10 and long half-life p25 in calcium-dependent manner [14, 15]. Similarly, the cleavage of p39 produces p29 and p10. Importantly, N-terminal p10 region assign of the two proteins underwent myristoylation on Gly2 determines their affinity to cell membrane, which signals for degradation. This can explain that the cleaved forms, p25 and p29 have long half-life than their precursors **(**Fig. 2**)**. Interestingly, Minegishi et al. swapped cognate p10 regions between p35 and p39, and found that p39 showed a slower degradation rate than p35 [15]. As a unique fragment of p35 or p39, p10 is necessary for the normal function of CDK5 complex in cells. A study showed that p10 protects against CDK5/p25-induced neurotoxicity by inhibiting both PRDX2 (Peroxiredoxin-2) phosphorylation and ROS accumulation in neurons [16]. On the contrary, p10 was reported to induce apoptotic morphologies via a caspases-independent pathway in cortical neurons [17], however, the detailed investigation on p10 in cancer development remains poorly reported.

Increasing studies showed that CDK5 plays an important role in cancer progress. Abnormal expression of CDK5 and its activators are positively associated with multiple tumorigenesis, as summarized in Fig. 1c**.** A recent study showed that high expression of CDK5 and p35 was observed in late stage of triple-negative breast cancer and correlated with the poor clinical outcome [18]. In glioblastoma, CDK5 expression was observed in 82.8% of WHO IV glioma [19]. Mechanistically, CDK5 can regulate cell growth, DNA repair and drug resistance in binding with p35 [20]. In contrast, as another activator, p39 expression was found to be decreased in 64% of human hepatocellular carcinoma, suggesting a tumor suppression role of p39 in HCC [21]. Interestingly, p39 expression is restricted to the postnatal brain [22], and thus its role is less reported in cancer. As shown in Fig. 1c, p39 is genetically deleted in multiple cancer types.

The allele frequencies of mutations on CDK5 are low in various cancer samples, but some mutations located in key domains of CDK5 likely influence its structure and PTMs, which contribute to tumorigenesis. As summarized in Table 1, R156H, A160T, V163G, R168H, P170S/L mutations located in T-loop of CDK5 with allele frequency of 0.1–0.42 were detected in tumor samples, and mutations R50W/Q, A48T and E50D were found in activators binding domain with allele frequency of 0.38–0.46, and G11W (allele frequency: 0.7) was mutated in ATP binding domain. Whether these mutations on CDK5 influence its PTMs requires more clinical and biological evidences.

**Table 1:** Summary of mutation sites in corresponding domain of CDK5 and their allele frequencies in clinical cancer samples

Cancer	Mutation	Mutation Type	Allele Frequency	Relative domain
Melanoma	P240L	Missense	0.21	Empty
	R156H	Missense	0.29	T-loop
	A196G	Missense	0.21	Empty
	P204L	Missense	0.58	Empty
	P204S	Missense	0.09	Empty
	G138V	Missense	0.12	Empty
	P100S	Missense	0.09	Empty
	X161splice	Splice	0.1	T-loop
	P100H	Missense	0.2	Empty
	X217splice	Splice	0.08	Empty
	L173F	Missense	0.2	Empty
Uterine	D73N	Missense	0.46	Empty
	R50W	Missense	0.29	ABD
	R168H	Missense	0.28	T-loop
	D99N	Missense	0.34	Empty
	R274H	Missense	0.09	Empty
	Q282H	Missense	0.19	Empty
	N62S	Missense	0.56	Empty
	R217Q	Missense	0.4	Empty
	A244T	Missense	0.09	Empty
	G113W	Missense	0.39	Empty
	N270D	Missense	0.04	Empty
	D92V	Missense	0.29	Empty
	D40Y	Missense	0.07	Empty
	F286L	Missense	0.33	Empty
	X265_splice	Splice	0.1	Empty
	F19L	Missense	0.13	Empty
	K237N	Missense	0.28	Empty
Sarcoma	A160T	Missense	0.16	T-loop
	D184N	Missense	0.12	Empty
Stomach	T221M	Missense	0.26	Empty
	P170S	Missense	0.27	T-loop
	R125M	Missense	0.13	Empty
	V64L	Missense	0.2	Empty
Lung adeno	I183F	Missense	0.23	Empty
	V249E	Missense	0.16	Empty
	G43S	Missense	0.25	Empty
Head & neck	A48T	Missense	0.24	ABD
	P170L	Missense	0.3	T-loop
	G138V	Missense	0.14	Empty
	P228L	Missense	0.36	Empty
Bladder	A31T	Missense	0.26	Empty
	L32V	Missense	0.07	Empty
Lung squ	A48T	Missense	0.38	ABD
Colorectal	R200W	Missense	0.11	Empty
	R200Q	Missense	0.34	Empty
	V163G	Missense	0.13	T-loop
	R50Q	Missense	0.35	ABD
	F91V	Missense	0.26	Empty
	G11W	Missense	0.7	ATP-BD
BICB	V162L	Missense	0.42	T-loop
	E101Q	Missense	0.73	Empty
	K61N	Missense	0.55	Empty
	E51D	Missense	0.46	ABD
	D99N	Missense	0.09	Empty
	K33Nfs*3	FS del	0.08	acetylation

## The biological function of CDK5 in cancer progression

Hyperactivation and overexpression of CDK5, as well as its activators p35 and p39, are frequently observed in colon cancer [23], breast cancer [24], lung cancer [25], thyroid cancer [26], pituitary adenoma [27] and prostate cancer [28], which regulate series events of cancer progression including proliferation, DNA damage response (DDR), apoptosis, migration, angiogenesis and immune evasion. During cancer development, p35 and p39 are upregulated in response to DNA damage. N-Myc (MYCN) transcriptionally binds to the promoter of p35 and p39 to promote their expressions [29]. Menin (MEN1) was reported as direct transcriptional factor of p35 to facilitate its expression, and therefore modulating synaptic plasticity [30]. During the differentiation of human leukemia to monocytes, EGR1 can increase the expression of p35 to activate CDK5 for cell differentiation [31]. In post-transcriptional level, microRNA-505-5p functions as a tumor suppressor by targeting CDK5 in cervical cancer [32] **(**Fig. 2**)**. These examples partially explain the co-occurrence of CDK5 and p35 frequently observed in cancer, more upstream regulators should be further investigated.

### The cellular distribution of CDK5

The cellular localization of CDK5 is closely related to its biological functions, and determined by its PTMs or the activity of p35 and p39. By forming complexes with p35 or p39, CDK5 can attach on cellular membrane or shuttle between cytoplasm and nucleus to exert its molecular functions. N-terminal p10 region of p39 and p35 containing localization motifs undergoes myristoylation that determines its membrane association, while removal of myristoylation on p39 or p35 can induce the nuclear localization of CDK5 [33]. Interestingly, CDK5 activity was reported to influence the cytoplasmic localization of p35-CDK5 and p39-CDK5 through phosphorylation of p35 or p39 on Ser8 **(**Fig. 2**)**. Inhibition of CDK5 kinase activity causes dephosphorylation and perinuclear accumulation of p35 or p39 [34]. It is estimated that approximately 25% of CDK5-p35 complexes are associated with membranes via ionic interactions together with lipidic interactions [35]. CDK5 can phosphorylate amphiphysin 1 on Ser276 and Ser285 to enhance its presence on lipid membrane for the regulation of synaptic vesicle endocytosis [36]. Another study showed that CDK5 localizes in the mitochondria-associated endoplasmic reticulum membrane (MAM), and regulates mitochondrial Ca^2+^ homeostasis. Deletion of CDK5 triggers mitochondrial permeability transition pore (mPTP) opening and mitochondrial Ca^2+^ transfer from the endoplasmic reticulum (ER) to the mitochondria [37]. More PTM events controlling the cellular localization of CDK5 remain further studied.

### PTM events in CDK5-mediated cancer proliferation

Cell cycle disorder and abnormal proliferation are two hallmarks of cancer cells. Major cell cycle regulators, such as CDKs, have been widely studied for their role in promoting cancer cell proliferation [38]. In addition, Noxa is constitutively expressed in cancer cells, and the CDK5 overexpression can promote the Noxa transfer from cytoplasm to mitochondria, participating in glucose metabolism to provide energy for nucleotide synthesis [39]. Huang et al. found that CDK5 is highly expressed in prostate, lung and breast cancer, which located in the nucleus and participates in the nuclear p21^CIP1^ protein degradation [40]. In non-small cell lung cancer (NSCLC), the positive staining of CDK5 and p35 was observed in the cytoplasm of malignant cells [41].

**Fig. 3:**
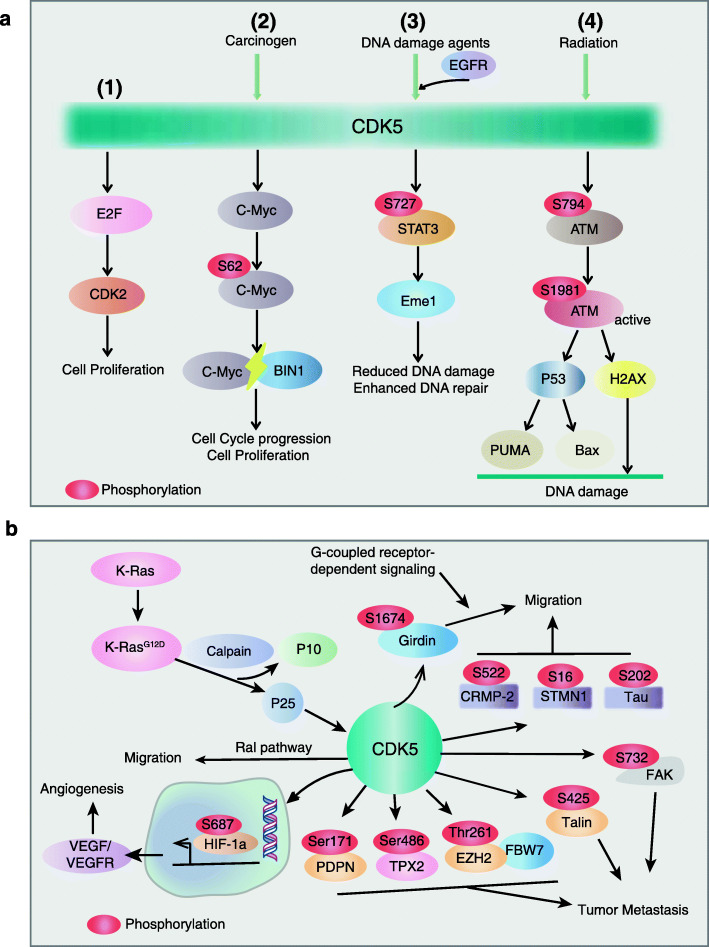
PTM events in CDK5-mediated biological function in cancer progression. **a** PTM events in CDK5-mediated cell proliferation and DNA damage repair. (1) In cancer cells, CDK5 mediates cell proliferation through activating E2F-CDK2 pathway. (2) CDK5 induces c-Myc Ser62 phosphorylation for abolishing Bridging integrator 1 (BIN1)/c-Myc interaction, promoting cell cycle progression. (3) During DNA damage, CDK5 can be up-regulated or phosphorylated by EGFR, inducing the phosphorylation of Ser727 on STAT3, the activated STAT3 promotes the expression of Eme1 gene to initiate DNA damage repair. (4) In response to radiation, ATM can be phosphorylated by CDK5 at Ser794 and undergoes autophosphorylation at Ser1981 for activation, which signals for p53 and H2AX pathways to initiate DNA damage repair. **b** The mechanism of CDK5 in apoptosis, cell migration and angiogenesis. CDK5 is activated upon EGF stimulation, which regulates downstream proteins phosphorylation, including Girdin, STMN1, CRMP-2, Talin and FAK to promote cell movement and migration. K-Ras^G12D^ promotes the production of p25 to enhance the activity of CDK5, which results in the activation of Ral (Ras-Like) pathway and morphological changes conducive to cell migration. In addition, CDK5 regulates angiogenesis through directly controlling the HIF1α target gene, VEGF and its receptor expression

As a proline-directed kinase, CDK5-mediated phosphorylation signaling cascade exhibits chief effect on multiple cancer progression **(**Fig. 3**,** Table 2**)**. In glioblastoma (GBM), CDK5 can amplify EGFR signaling by phosphorylating CRMP2 (collapsin response mediator protein 2) at Ser522, sustaining the pro-proliferation effect on tumor cells [46]. Retinoblastoma protein (Rb), a key cell cycle regulator that binds to E2F for preventing cell proliferation, was reported to be a downstream substrate of CDK5 [47]. In medullary thyroid, CDK5 activation can enhance the expression of E2F downstream target genes CDK2, p15^INK4b^ and p21^CIP/WAF1^ to promote cell proliferation **(**Fig. 3a**)**. It phosphorylates Rb at Ser807/Ser811 to release transcription factor E2F for initiating cell cycle [26]. In addition, CDK5-mediated c-Myc phosphorylation at Ser62 can abolish Bridging integrator 1 (BIN1)/c-Myc interaction, ultimately facilitating the progression of NSCLC [48]. Recent study showed that CDK5 activated by EGFR can phosphorylate TRIM59 (Tripartite motif-containing 59) at Ser308, which recruits PIN1 (Peptidylprolyl Cis/Trans Isomerase, NIMA-Interacting 1) for TRIM59 cis-trans isomerization. The isomerized TRIM59 undergoes a nucleus translocation via binding to importin α5, and enhances STAT3 signaling for promoting tumorigenesis by inducing ubiquitination and degradation of tumor suppressor histone variant macroH2A1 [49].

**Table 2:** CDK5 mediated phosphorylation events in cancers

Cancers	Protein	Positions	Signal pathways	References
Pancreatic	K-Ras	Gly12 mutant Asp12	MEK, PI3K, CDK5 signaling and regulatory pathways	[42]
Medullary thyroid carcinoma	STAT3	Ser727 phosphorylation	STAT3 pathway	[43]
Non-small cell lung	c-Myc	Ser62 phosphorylation	c-Myc pathway	[44]
Small cell lung	c-Myc	Ser62 phosphorylation	c-Myc pathway	[44]
Colorectal	ERK5	Thr732 phosphorylation	ERK5-AP-1 pathway	[23]
Breast	FAK	Ser732 phosphorylation	CDK5-FAK pathway	[24]
Glioblastoma multiforme	PIKE-A	Ser279 phosphorylation	PIKE-A-Akt pathway	[45]

### PTM events in CDK5-mediated DNA damage response and DNA repair

During tumor progression, a decrease of damaged surveillance mechanism and an enhancement of genome instability are necessary for cancer cells to achieve uncontrolled growth and the adaptability associated with aggressive tumors [50]. Deregulation of DNA damage response (DDR) plays an important role in cancer development [50–52]. CDK5 was reported to participate in the DDR process mainly by phosphorylating series major DDR proteins **(**Fig. 3a**)**, such as ATM (ataxia-telangiectasia mutant) and Ape1 (apurinic/apyrimidine endonuclease 1) [53, 54]. The inhibition of CDK5 kinase activity was linked to suppression of DDR process and tumor progression. Courapied and the colleagues found that CDK5 can influence the DDR process by up-regulating the basic meiotic structure-specific Eme1 (Essential Meiotic Structure-Specific endonuclease 1) mediated by STAT3 [55]. DNA damage stimulation leads to the up-regulation of CDK5, which induces the phosphorylation of Ser727 on STAT3 to activate STAT3, indicating that CDK5-STAT3-Eme1 signaling plays a critical role in DNA damage repair (Fig. 3a**)**.

Many traditional chemotherapeutic agents are DNA damage stimulators, for instant, ionizing radiation and topoisomerase inhibitors can enhance the activity of CDK5 in cancer cells. CDK5 is able to induce the phosphorylation of ATM at Ser729, to activate DNA damage repair to promote cancer proliferation [56]. In addition, knockdown of CDK5 can decrease the cellular DNA damage repair ability and inhibit tumor cell growth [57], suggesting that inhibition of CDK5 in combination with traditional chemotherapeutic agents may be an effective strategy for cancer treatment.

### Dual role of CDK5 PTMs in apoptosis

The dual role of CDK5 in apoptosis was found in different diseases, depending on the occurrence of PTMs at specific proteins/sites. Hsu et al. found that CDK5/p35 complex can induce the Ser81 phosphorylation of AR (androgen receptor) to inhibit cell apoptosis and promote cell proliferation in prostate cancer [58]. Recent study showed that CDK5-mediated Mcl-1 phosphorylation can lead to the inactivation of the apoptosis signal pathway in PDAC (Pancreatic ductal adenocarcinoma) [59]. The above observations demonstrated that CDK5-mediated PTM events play a critical role in controlling apoptosis.

### PTMs on CDK5 for cancer metastasis

Metastasis is a key feature in cancer progression, and the activity of CDK5 was found to be associated with invasive phenotype, such as cytoskeleton remodeling [60]. A study showed that the mutant form of K-Ras^G12D^ promotes the generation of p25 that further enhances the activity of CDK5 in pancreatic cancer, resulting in the activation of Ral (Ras-Like) pathway and morphological change conducive to cell migration [42, 60, 61]. In breast cancer, EGF (epidermal growth factor) stimulation activates CDK5 to phosphorylate Girdin (Gα-interacting vesicle associated protein) at Ser1674, which activates the downstream G-coupled receptor-dependent signaling pathway for promoting cell migration [62] **(**Fig. 3b**)**. Increasing evidences proved that CDK5 is involved in the regulation of the microtubule complex that is necessary for cell movement. For example, the Ser202 phosphorylation of microtubule-associated protein Tau [63], the Ser522 phosphorylation of CRMP-2 (Collapsin Response Mediator Protein-2) [64] and the Ser16 phosphorylation of STMN1 (stathmin, microtubule decomposing protein) [65] are all CDK5 substrates that associated with carcinogenesis and tumor migration. Furthermore, CDK5 can promote tumor migration through the Ser425 phosphorylation of talin and the Ser732 phosphorylation of FAK (Focal adhesion kinase) [66] **(**Fig. 3b**)**. Jin et al. found that CDK5 regulates Thr261 phosphorylation in EZH2 (Enhancer of zeste homolog 2), a component of the polycomb repressive complex 2 (PRC2). The phosphorylated EZH2 can bind to FBW7 (F-box and WD repeat domain-containing 7) for degradation to prevant pancreatic cell migration and invasion [67]. In addition, CDK5 can inhibit mouse cell migration through Ser171 phosphorylation in PDPN (Podoplanin) [68]. Recent studies found that CDK5-mediated phosphorylation of TPX2 (Target protein for Xklp2) at Ser486 stabilizes TPX2 to promote the migration of hepatocellular carcinoma cells [69] **(**Fig. 3b**)**. Collectively, CDK5-mediated phosphorylation cascade is critical for tumor metastasis, suggesting that targeting CDK5 is a promising strategy for preventing cancer spread.

### PTM events in CDK5-mediated tumor angiogenesis

Angiogenesis is the physiological process of growing new blood vessels from existing blood vessels, which provides nutrition and oxygen for tumor growth. Julia Herzog et al. showed that CDK5 is the main regulator of angiogenesis in hepatocellular carcinoma (HCC). CDK5 can directly phosphorylate HIF1α (hypoxia inducible factor 1α) at Ser687 to enhance the expression of its target genes VEGFA, VEGFR1 and EphrinA1, which are essential for the formation of novel blood vessels in tumors [70] **(**Fig. 3b**)**. In U87 glioblastoma and Lewis lung cancer, inhibition of CDK5 can reduce VEGF expression to inhibit angiogenesis [71]. Taken together, CDK5 is also a potential target for inhibiting angiogenesis in cancer therapy.

### PTM events in CDK5-mediated immune evasion

Immune evasion often occurs through the mechanism of peripheral tissue tolerance in cancers, for example, inhibiting the expression of programmed cell death ligand 1 (PD-L1) can produce potent anti-tumor immunity [72]. Recent study showed that CDK5 has the function of regulating PD-1/PD-L1 pathway in immunity. Dorand et al. found that CDK5 can directly or indirectly inhibit the activity of phosphorylase to inhibit regulatory factor 2 binding protein 2 (IRF2BP2) at Ser360 phosphorylation, which further leads to tumorigenesis [73]. Simultaneously, they found that inhibition of CDK5 activity can regulate the continuous expression of interferon regulatory factor 2 (IRF2) and interferon IRF2BP2 in medulloblastoma (MB) mouse model, decreasing PD-L1 expression and then eliminating immune evasion [72]. In addition, aPBAE/Cas9-CDK5 nanoparticles developed by Huan Deng et al. can effectively knock out CDK5 in vitro, resulting in down-regulation of PD-L1 expression and inhibition of tumor growth [74].

## Various types of PTMs on CDK5

With the advanced mass spectrometric technology developed, new PTMs on CDK5 including glycosylation, phosphorylation, ubiquitin, sumoylation and acetylation were identified to play important roles in tumorigenesis [75–77]. These PTMs are found to be essential for modulating the activity of CDK5 during tumor occurrence and development. The new discovery of PTM sites on CDK5 is shown in Table 3 and their biological functions are outlined as follows.

**Table 3:** PTMs on CDK5 and their relevant functions

Positions	PTM types	Molecular Functions
Thr14	Phosphorylation	Decreases CDK5 activity
Tyr15	Phosphorylation	Increases CDK5 activity and promotes cell growth
Lys33	Acetylation	Decreases CDK5 activity by inducing CDK5 loss ATP binding ability
Lys56	Acetylation	Unknown
Cys83	S-nitrosylation	Increases CDK5 activity; Regulates the development of neurons
Cys157	S-nitrosylation	Increases CDK5 activity; Regulates the development of neurons
Ser159	Phosphorylation	Decreases CDK5 activity kinase

### Phosphorylation events relevant to CDK5

CDK5 phosphorylation and CDK5-mediated phosphorylation events were reported to play critical roles in degenerative neurological diseases and cancer [13]. Phosphorylation on CDK5 is the most widely investigated PTM that contributes to the activity of CDK5. Zukerberg et al found that c-Abl can induce Tyr15 phosphorylation of CDK5 to promote neurite outgrowth [78]. Ehrlich and colleagues found that Tyr15 phosphorylation of CDK5 and p35 is upregulated in human hepatoblastoma cell lines as compared to primary human hepatocytes [56]. In GBM tumorigenicity, Tyr15 phosphorylation of CDK5 activated by EGFR phosphorylates TRIM59 at Ser308 to promote tumor growth [49]. The activity of CDK5 is inhibited by phosphorylation of Thr14, since the phosphorylation causes the misalignment of the ATP-phosphate group and the change of the Mg^2+^ ion coordination sphere, as well as the G-loop to shift away from the ATP binding site [8]. Phosphorylation of Ser159 on CDK5 was also reported to display an inhibitory role in the activity of CDK5 kinase [9].

For CDK5-mediated phosphorylation events, its substrates are involved in a variety of biological processes regulation and are closely related to the occurrence and development of cancer. For example, the activity of CDK5 during neuronal development can be realized by phosphorylation of p35 at Thr138 position [79, 80]. Recent study found that, as a downstream target of CDK5, Rb is phosphorylated by CDK5, eventually increasing the expression of cyclin and other CDKs [26]. On the other hand, inhibiting CDK5 activity can decrease calmodulin Tyr27 phosphorylation, thereby reducing melanoma cell cytoskeleton remodeling, metastasis and invasion [61]. Furthermore, CDK5 enhances PI3K/AKT signal transduction by phosphorylation of Gα–interacting vesicle associated protein at Ser1674 position, which leads to cell migration [62].

### S-nitrosylation of CDK5

S-nitrosylation refers to the addition of NO group to a cysteine residue of certain proteins, which plays diverse regulatory roles in multiple physiological processes. Its role in cancer development was widely investigated in recent years. In breast cancer, S-nitrosylation of Ras induces MAPK-dependent phosphorylation and activates ETS-1 (Erythroblastosis virus transcription factor-1), a critical mediator of nitric oxide, resulting in an aggressive breast cancer phenotype [81]. S-nitrosylation of H-Ras also restricts Raf-1 activation and further signals propagation via ERK-1/2 [82].

**Fig. 4:**
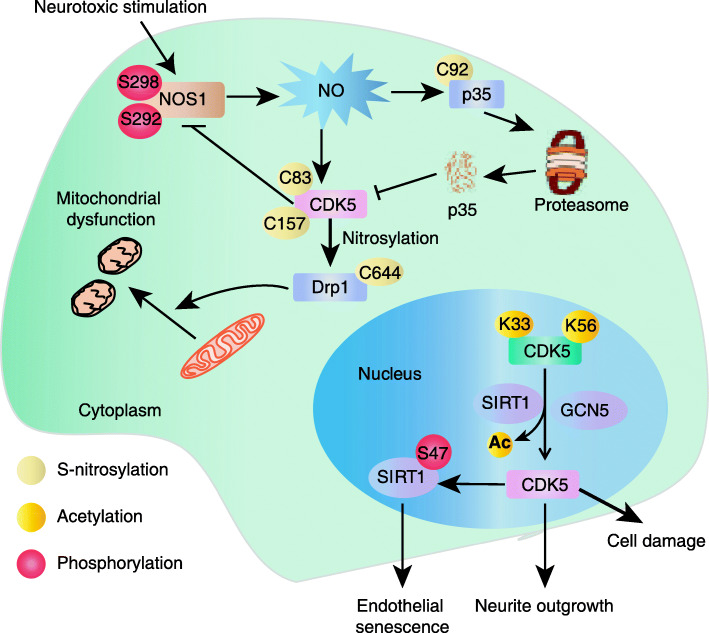
S-nitrosylation and acetylation of CDK5 in cell. The interaction between NOS1 and CDK5 promotes the formation of SNO-CDK5, and then SNO-CDK5 may transfer NO group to DRP1 by transnitrosation, resulting in excessive mitochondrial division and subsequently mitochondrial dysfunction. SNO-CDK5 phosphorylates NOS1 on S292 and S298, creating a negative-feedback loop by suppressing NOS1 activity. In addition, NO is able to negatively regulate the activity of CDK5 by inducing p35 S-nitrosation at C92 for controlling the development of neuronal cells. In nucleus, the acetylation of CDK5 at K33 and K56 mediated by SIRT1 and GCN5 leads to the loss of ATP binding and the impairment of kinase activity, which regulates multiple cellular processes, including neurite outgrowth and cell damage. The activated CDK5 can in turn phosphorylate SIRT1 at S47 that contributes to cell senescence

In CDK5, Cys83 and Cys157 can be S-nitrosylated, which leads to overactivation of CDK5 and subsequently contributes to mitochondrial dysfunction, synaptic damage and neuronal cell death. A study showed that the interaction between neuronal NOS1 (nitric oxide synthase) and CDK5 contributes to the formation of SNO-CDK5 [83]. Subsequently, SNO-CDK5 transfers the NO group to Drp1 (dynamin-related protein 1) through trans-nitrosation, the formation of S-nitroso-Drp1 leads to excessive mitochondrial division and synaptic failure [84]. In turn, hyperactivated CDK5 phosphorylates NOS1 and suppresses its activity in a negative regulatory-feedback loop [85] **(**Fig. 4**)**. On the contrary, zhang et al. proved that the mutation of Cys83 S-nitrosylation on CDK5 restores its kinase activity and enhances dendritic growth and branching [86]. NO acts as a molecular switch to negatively regulate the activity of CDK5 in a p35 S-nitrosation-dependent way for controlling the development of neuronal cells [87]. Considering the fact that CDK5 phosphorylates many target substrate proteins, it is possible that CDK5 may also nitrify these substrates to influence multiple biological progresses [7]. In addition, the imbalance of CDK5 activity may play a role not only in AD (Alzheimer’s disease), but also in several neurodegenerative diseases. However, the S-nitrosylation of CDK5 involved in tumorigenesis remains to be studied, thus it is interesting to investigate the role of SNO-CDK5 in cancer development.

### Sumoylation and acetylation on CDK5

Sumoylation is involved in cell biological processes such as cell cycle regulation, senescence, and apoptosis [88, 89]. Recent experiments showed that the deregulation of the sumo pathway promotes carcinogenic transformation by affecting (de) sumoylation of many oncoproteins and tumor suppressors [90]. Sumoylation on the complex of CDK5/p35 is associated with the activity of CDK5 kinase [91]. Anja Büchner et al. used co-immunoprecipitation assay to investigate the effect of sumoylation on CDK5/p35 complex formation. They found that p35 is a novel sumoylation target, in which p35 sumoylation can enhance the formation of CDK5/p35 complex [92], however, it is not clear whether sumoylation can directly act on CDK5. The details for how the sumoylation of CDK5 is involved in regulating the occurrence and development of tumor remain to be studied.

K33 and K56 are two acetylation sites of CDK5 that were firstly identified by Juhyung Lee et al. in 2014 [93]. They found that the acetylation of K33 on CDK5 in the nucleus can lead to the loss of ATP binding ability and the destruction of kinase activity [94]. In addition, their experiments further demonstrated that GCN5 (General control of amino acid synthesis protein 5) and SIRT1 (Sirtuin-1) are the key factors for deacetylation of CDK5. Thus, SIRT1 inhibitors can enhance acetylation of CDK5 for decreasing its activity **(**Fig. 4**)**. However, the biological function of K56 of CDK5 remains to be studied [95, 96]. Interestingly, hyperactivation of CDK5 is able to phosphorylate SIRT1 at S47 to influence cellular senescence [97]. Regarding the functional role of the acetylation modification of CDK5 in neurological diseases, and whether CDK5 also plays a similar role in the development of cancer require further investigations.

## Targeting CDK5 and its PTMs as potential cancer therapies

### Clinical trials of CDK inhibitors in cancer therapy

Hyperactivation of CDKs was frequently recorded in most primary tumors, and thus were deemed as useful targets for clinical cancer therapy. Since CDK activity relies on ATP, most of the current CDKs inhibitors are based on targeting ATP binding pocket. For example, flavopiridol, the first pan-CDK inhibitor used in human clinical trials, can bury in the ATP-binding pocket of CDKs, and inhibits their activity at nanomolar dosage [98]. Several phase I clinical trials showed that flavopiridol has an antitumor effect in patients with renal, prostate and colon cancer [99]. In the past decade, many classes of CDK inhibitors were under investigation in clinical trials, such as olomoucine, roscovitine, kenpaullone and SNS-032, which achieved significant therapeutic significance in clinic. Another successful example is CDK4/6 inhibitors, three CDK4/6 inhibitors including palbociclib, ribociclib and abemaciclib have currently received FDA approval for clinical use [100]. Mechanically, these inhibitors can inhibit Rb protein phosphorylation and arrest the cell cycle in G1 phase to inhibit proliferation [100]. Though the PTMs on CDKs are essential for their functions, the inhibitors that specifically modify PTMs of CDKs are lacking in clinical trials.

### The inhibitors of CDK5 in cancer

Considering the oncogenic role of CDK5 in cancer, targeting the ATP binding of CDK5 and CDK5-p35 interaction is currently the two major strategies to suppress CDK5 activity, which has been used in preclinical trials of drugs for several types of tumors (Table 4) [114–116]. Under phase II clinical trials, dinaciclib (SCH 727965) is an ATP-competitive inhibitor used to inhibit CDK5 activity [105, 117], it displays 10-fold higher therapeutic index than flavopiridol [105, 118–120] with a range of potency and pharmacokinetic parameters. In addition, small molecules like PHA-767491 and PHA793887 were reported to inhibit the activity of CDK5 by occupying the ATP binding sites of CDK5, exerting an anticancer effect on cervical cancer and breast cancer [101–104]. The ATP binding pocket on CDK5 is structurally conserved, and thus many ATP competitive inhibitors of CDK5 can nonspecifically bind with other CDKs [121].

**Table 4:** Summary of CDK5 inhibitors

Inhibitors	Type	Major Targets	Disease(s)	References
PHA-767491	Drug like	CDK1, CDK2, CDK5, CDK9	Cervical; Breast	[101, 102]
PHA-793887	Drug like	CDK1, CDK2, CDK4, CDK5, CDK7, CDK9	Myeloma; Lung	[103, 104]
Flavopiridol	ATP-competitive	CDK1, CDK2, CDK5, CDK9	Retinoblastoma protein	[105]
Dinaciclib (SCH727965)	ATP-competitive	CDK1, CDK2, CDK5, CDK9	Prostate	[105]
Roscovitine (Seliciclib, CYC202)	ATP-competitive	CDK2, CDK5	Liver; Lymphoma	[106]
Hymenialdisine	ATP-competitive	CDK5, GSK3β, CDK2, CDK1, Chk1	Alzheimer’s disease	[107]
Purvalanol-A	ATP-competitive	CDK1, CDK2, CDK5	Breast	[108]
Indirubin-5	ATP-competitive	CDK1, CDK2, CDK5	Leukemia	[109]
AT7519	ATP-competitive	CDK2, CDK4, CDK5, CDK9	Colon; Leukemia	[110]
CIP (peptide derived from p53)	Peptide competing with Substrate	CDK5/p25	Pancreatic	[111]
CIP (peptides derived from p35)	Peptide competing with Substrate	CDK5/p35	Alzheimer’s disease	[112]
CPD1-3α-amino-5α androstane	Small molecule non-ATP competitive	CDK5/p35	Skin cancer	[113]

Another class of CDK5 inhibitors can inhibit the CDK5 activity by abolishing the interaction of CDK5/p35 complex. Roscovetine (Seliciclib, CYC202) was reported to inhibit the activity of CDK5 by competing for the binding site of p35 [106]. Tamoxifen can compete for binding with p35 and p25 to inhibit the CDK5 activity in breast cancer [69]. Besides, CDK5 inhibitory peptide (CIP), a polypeptide containing 125 amino acid residues derived from the peptide sequence 154-279aa of CDK5 activating protein p35, has a specific inhibitory activity on CDK5. CIP can bind to CDK5/p25 to form a ternary complex, blocking the interaction between CDK5 and p25 protein and thus inhibiting the activity of CDK5. A study showed that CIP can inhibit the abnormal phosphorylation of Tau induced by CDK5/p25 [111]. Medicinal polypeptides and the FDA drugs, such as tamoxifen, exhibit both low side-effects and significant anticancer effects; however, their stability and bioavailability remain the shortcoming for clinical application, and maybe natural product source inhibitors are more compatible to achieve higher therapeutic effect [122, 123]. Lastly, as mentioned above, the activity of CDK5 is mainly controlled by various PTMs. For example, Ser159 phosphorylation and Lys33 acetylation negatively regulate the CDK5 activity, while Tyr15 phosphorylation links to CDK5 hyper-activation. It seems that targeting these PTM modifiers directly or indirectly could be a promising strategy for the inhibition of CDK5 activity and thus for the chemoprevention of cancer.

## Conclusion and outlook

CDK5 is considered to be an atypical member of the CDKs, and its abnormal expression is involved in a variety of diseases, such as neurodegenerative diseases and cancer. During cancer progression, CDK5 serves as an oncogene to promote cell proliferation, migration and invasion. Currently, emerging types of PTM on CDK5 were identified, and were found to be involved in a wide range of cellular processes in cancer. ATP competitive inhibitors and drugs by targeting CDK5-p35 complexes are currently the major inhibitory tools to reduce CDK5 activity, however, their low specificity/stability and high toxicity remain handicap for clinical application. Increasing clinical studies have found that the CDK5 activity inhibitors are not specific, since they inhibit the activity of other kinases as well (Table 4). In this regard, specifically targeting PTMs on CDK5 may be a promising strategy for cancer treatment. This is possible because novel and rare PTMs that may significantly regulate the activity of CDK5 will be identified by employing the advancing technology of mass spectrometry and other analytical methods. The data reviewed above provide robust evidences to propose the inclusion of CDK5 and its PTMs in the group of novel molecules to be tested in preclinical research aiming at tumor intervention. The association of CDK5 inhibitors with the large number of available drugs currently under investigation is likely to offer additional rational therapeutic approaches for cancer.

## Data Availability

All the data obtained and/or analyzed during the current study were available from the corresponding authors on reasonable request.

## Abbreviations

ABD: Activators binding domain
ACC: Adrenocortical carcinoma
AD: Alzheimer’s disease
AML: Acute myeloid leukemia
AEP1: Apurinic/apyrimidine endonuclease 1
AR: Androgen receptor
ATM: Ataxia-telangiectasia mutant
ATP-BD: ATP binding domain
BICB: Breast invasive carcinoma breast
BIN1: Bridging integrator 1
CCCs: Cholangiocarcinoma
CDK5: Cyclin-dependent kinase 5
CDKs: Cyclin-dependent kinases
CHK1: Checkpoint kinase 1
CIP: CDK5 inhibitory peptide
CPD1: Cercosporin photosensitizer detoxification
ccRCC: Clear cell renal cell carcinoma
CRMP2: Collapsin Response Mediator Protein 2
DDR: DNA damage response
DLBC: Lymphoid neoplasm diffused large b-cell lymphoma
Drp1: Dynamin-related protein 1
EGF: Epidermal growth factor
EGR1: Early growth response-1
Eme1: Essential Meiotic Structure-Specific Endonuclease 1
ER: Endoplasmic reticulum
ETS-1: Erythroblastosis virus transcription factor-1
EZH2: Enhancer of Zeste homolog 2
FAK: Focal adhesion kinase
FBW7: F-box and WD repeat domain-containing 7
GBM: Glioblastoma
GCN5: General control of amino acid synthesis protein 5
Girdin: Gα-interacting vesicle associated protein
GSK-3β: Glycogen synthase kinase-3β
HCC: Hepatocellular carcinoma
HIF1α: Hypoxia inducible factor 1α
IRF2: Interferon regulatory factor 2
IRF2BP2: Inhibit regulatory factor 2 binding protein 2
LGG: Lower grade glioma
Lung adeno: Lung adenocarcinoma
Lung squ: Lung squamous cell carcinoma
MAM: Mitochondria-associated endoplasmic reticulum membrane
MB: Medulloblastoma
MEN1: Menin
mPTP: Mitochondrial permeability transition pore
N-Myc: Neuroblastoma MYC Oncogene
NOS1: Nitric oxide synthase 1
Noxa: Nitric oxide synthase
PMAIP1: Phorbol-12-myristate-13-acetate-induced protein 1
NSCLC: Non-small cell lung cancer
p35: Cyclin dependent kinase 5 regulatory subunit 1, CDK5R1
p39: Cyclin dependent kinase 5 regulatory subunit 2, CDK5R2
PCPG: Pheochromocytoma and paraganglioma
PDAC: Pancreatic ductal adenocarcinoma
PD-L1: Programmed cell death ligand-1
PDPN: Podoplanin
PIN1: Peptidylprolyl cis/Trans isomerase NIMA-Interacting 1
PRC2: Polycomb repressive complex 2
pRCC: Papillary renal cell carcinoma
PRDX2: Peroxiredoxin-2
PTMs: Posttranslational modifications
Ral: Ras-Like
Rb: Retinoblastoma protein Reactive oxygen species
STMN1: Stathmin, microtubule decomposing protein
SIRT1: Sirtuin-1
Tau: Microtubule-associated protein Tau
TPX2: Target protein for Xklp2
TRIM59: Tripartite motif-containing 59
Uterine CS: Uterine carcinosarcoma
UM: Uveal melanoma
